# Tripterine Liposome Exerts Antitumor and Immunomodulatory Effects in Pancreatic Ductal Adenocarcinoma

**DOI:** 10.1002/mco2.70977

**Published:** 2026-09-20

**Authors:** Lirui Tang, Yi Lin, Xueqin Jiang, Xiaohe Tian, Giuseppe Battaglia, Yuquan Wei, Wenshuang Wu, Xuemei He, Xiawei Wei

**Affiliations:** ^1^ Laboratory of Aging Research and Cancer Drug Target State Key Laboratory of Biotherapy and Cancer Center National Clinical Research Center for Geriatrics West China Hospital Sichuan University Chengdu China; ^2^ Department of Biotherapy Cancer Center and State Key Laboratory of Biotherapy West China Hospital Sichuan University Chengdu China; ^3^ Department of Radiology Functional and Molecular Imaging Key Laboratory of Sichuan Province Huaxi MR Research Centre (HMRRC) Chengdu China; ^4^ Institute for Bioengineering of Catalonia (IBEC) The Barcelona Institute of Science and Technology Barcelona Spain; ^5^ Division of Thyroid Surgery Department of General Surgery and Laboratory of Thyroid and Parathyroid Disease Frontiers Science Center for Disease‐related Molecular Network West China Hospital Sichuan University Chengdu China

**Keywords:** liposome, pancreatic ductal adenocarcinoma, tripterine, tumor microenvironment

## Abstract

Pancreatic ductal adenocarcinoma (PDAC) is characterized by a highly aggressive phenotype and limited therapeutic options, primarily attributable to diagnostic delays and intrinsic chemotherapy resistance, thereby underscoring the critical need for the development of more effective therapeutic strategies. Tripterine (TP) is a natural compound with potent antitumor activity, but has shown limited clinical use due to its multi‐organ toxicity. Here, we overcame this limitation by formulating TP into liposomes (TP‐lipo). We evaluated the therapeutic potential of TP‐lipo showing that TP‐lipo exhibited antitumor activity in vitro (murine *Pdx1‐Cre*/*LSL‐Kras^G12D/+^*/*LSL‐Trp53^R172H/+^* pancreatic cancer cells, KPC cells) and in vivo (PDAC mouse) models. TP‐lipo reshaped the tumor immune microenvironment by promoting macrophage polarization toward the M1 phenotype, increasing the proportion of interferon‐γ (IFN‐γ)‐secreting CD8^+^ T cells. In vitro, TP‐lipo promoted IFN‐γ and granzyme B production in CD8^+^ T cells, while TP‐lipo‐pretreated macrophages enhanced T‐cell differentiation into cytotoxic T lymphocytes. Notably, combination therapy with TP‐lipo and PD‐1 antagonist exerted a synergistic antitumor effect and significantly prolonged median survival in PDAC‐bearing mice. TP‐lipo showed substantially lower toxicity than the free drug, highlighting its improved safety profile and therapeutic potential. Collectively, these findings suggest TP‐lipo, particularly in combination with PD‐1 blockade, as a promising therapeutic strategy for PDAC.

## Graphical abstract

Tripterine liposome (TP‐lipo) exerts anti‐pancreatic ductal adenocarcinoma (PDAC) effects through direct suppression of tumor cells and modulation of the tumor microenvironment (TME). TP‐lipo induces apoptosis and inhibits the clonogenicity, migration, and invasion of KPC cells in vitro. In parallel, it reshapes the immunosuppressive TME by promoting macrophage polarization toward the antitumor M1 phenotype and enhancing granzyme B and interferon‐gamma (IFN‐γ) production by CD8^+^ T cells. TP‐lipo‐polarized macrophages further reinforce CD8^+^ T‐cell effector function, collectively contributing to PDAC suppression.

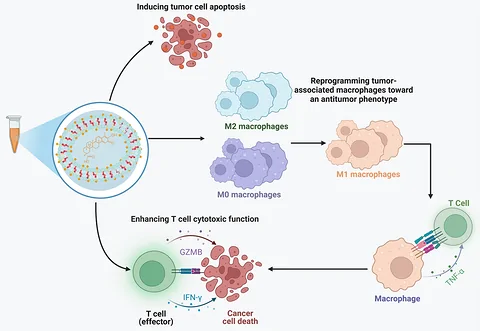

## 1 Introduction

Pancreatic cancer is highly lethal, with mortality rates nearly matching its morbidity rates [1]. Across all disease stages, patients exhibit a median survival time of approximately 4 months and a 5‐year survival rate of only 13% [2]. Pancreatic ductal adenocarcinoma (PDAC) presently ranks as the seventh leading cause of cancer‐related mortality globally. Projections indicate that this figure will increase by over 80% by 2040. This upward trend is especially evident in high Human Development Index (HDI) regions [3]. Notably, in countries such as the United States, where mortality rates from other cancers are declining due to improved screening and treatment, PDAC is still expected to become the second leading cause of cancer‐related deaths by 2030 [4]. This poor prognosis mainly stems from the early systemic dissemination of tumor cells and the inadequate efficacy of systemic treatments, making systemic disease control extremely difficult, even in patients initially presenting with localized disease on imaging [2]. Moreover, PDAC exhibits strong resistance to immunotherapies due to immunosuppressive interactions among immune cells, driven by the highly immunosuppressive nature of its tumor microenvironment (TME) [5]. Characterized as “cold” TME, PDAC is marked by extensive infiltration of pro‐tumor myeloid cells and few effector CD8^+^ T cells, which are critical for antitumor immunity [6].

The development and maintenance of the highly immunosuppressive TME in pancreatic cancer involve complex interplay among multiple molecular and cellular networks. For instance, focal adhesion kinase (FAK) is overexpressed in human PDAC tissues, and its high expression strongly correlates with increased TME fibrosis and insufficient infiltration of cytotoxic CD8^+^ T cells [7]. Additionally, tumor cell–derived prostaglandin E2 (PGE2) and tumor necrosis factor (TNF) induce the expansion of a pathogenic IL‐1β^+^ macrophage subset, which drives a self‐reinforcing loop that reprograms pancreatic cancer cells toward aggressive, immune‐evasive phenotypes [8]. Consequently, targeting the TME to reverse its immunosuppressive property is a crucial therapeutic strategy. Proline‐rich tyrosine kinase 2 (PYK2) functions as an “immunomechanical” checkpoint by sensing tumor stiffness and translating these physical signals into immune responses [9]. Consequently, the knockdown of PYK2 inhibits monocyte‐to‐macrophage differentiation, reprograms the PDAC TME, and enhances the efficacy of anti‐PD‐1 therapy [9].

Tripterine (TP), a prominent bioactive triterpenoid derived from *Celastraceae* family members such as *Tripterygium wilfordii* Hook F, is widely recognized for its therapeutic potential [10]. Beyond its roles in treating inflammatory, cardiovascular, and neurodegenerative disorders, TP also demonstrates potent antitumor activity across a variety of malignant tumors, including ovarian, lung, gastric cancers, multiple myeloma, osteosarcoma, and pancreatic cancer [11]. Its antitumor mechanisms are multifaceted, involving direct inhibition of tumor cell proliferation, induction of apoptosis and immunogenic cell death (ICD), and regulatory effects on the TME. Both in vitro and in vivo studies have demonstrated that TP repolarizes tumor‐associated macrophages from an M2 to an M1 phenotype in the colorectal cancer model [12]. In melanoma models, TP remodels the obesity‐related immunosuppressive TME by inducing ICD, restoring T‐cell metabolic fitness via upregulation of prolyl hydroxylase‐3, and enhancing T‐cell infiltration [13]. In PDAC, it was reported that TP covalently binds to the Cys194 residue of the sorcin protein to disrupt the sorcin‐paired box 5 (PAX5) interaction, triggers PAX5 nuclear import to upregulate F‐box and leucine‐rich repeat protein 12, and thereby induces ferroptosis in pancreatic cancer cells [14]. However, its clinical translation is severely limited by multi‐organ toxicity and extremely poor aqueous solubility. Studies in lipopolysaccharide (LPS)‐induced mouse sepsis models revealed that TP caused hepatic morphological damages and oxidative stress, and upregulated the expression of renal tubular injury markers such as kidney injury molecule‐1 and neutrophil gelatinase‐associated lipocalin (NGAL) [15]. Additionally, TP inhibits human ether‐a‐go‐go‐related gene (hERG) channel activity in human embryonic kidney 293 cells, inducing cardiotoxicity [16]. For bioavailability, only 0.044 mg of TP can be dissolved in 1 mL of solvent at 25°C [17]. Although various TP delivery platforms, including nanostructured lipid carriers, dendrimer conjugates, and PEG‐PLGA/PEG‐PCL micelles [18], have been developed to enhance its solubility, reduce toxicity, and improve therapeutic efficacy in preclinical models, few have been validated in PDAC. Consequently, there remains a need for effective formulations that can directly target tumor cells and remodel the TME in PDAC.

In this study, we developed a liposomal TP formulation (TP‐lipo) and investigated its therapeutic effects in mouse models of PDAC. Our results showed that TP‐lipo significantly reduced tumor burden in mice and demonstrated prominent antitumor activity. Mechanistically, TP‐lipo could directly induce apoptosis, suppress the invasiveness and clonogenicity of KPC cells, and modulate the TME by regulating macrophage polarization and enhancing cytotoxic CD8^+^ T cells. We further evaluated the combined therapeutic effects of TP‐lipo and PD‐1 antibody (aPD‐1) in mouse PDAC models. The results revealed that combination therapy significantly extended median survival compared to either monotherapy. Importantly, TP‐lipo exhibits reduced toxicity and improved safety compared to free TP, offering the potential to expand the therapeutic toolkit for PDAC.

## 2 Results

### 2.1 Characterization of TP‐lipo

**FIGURE 1:**
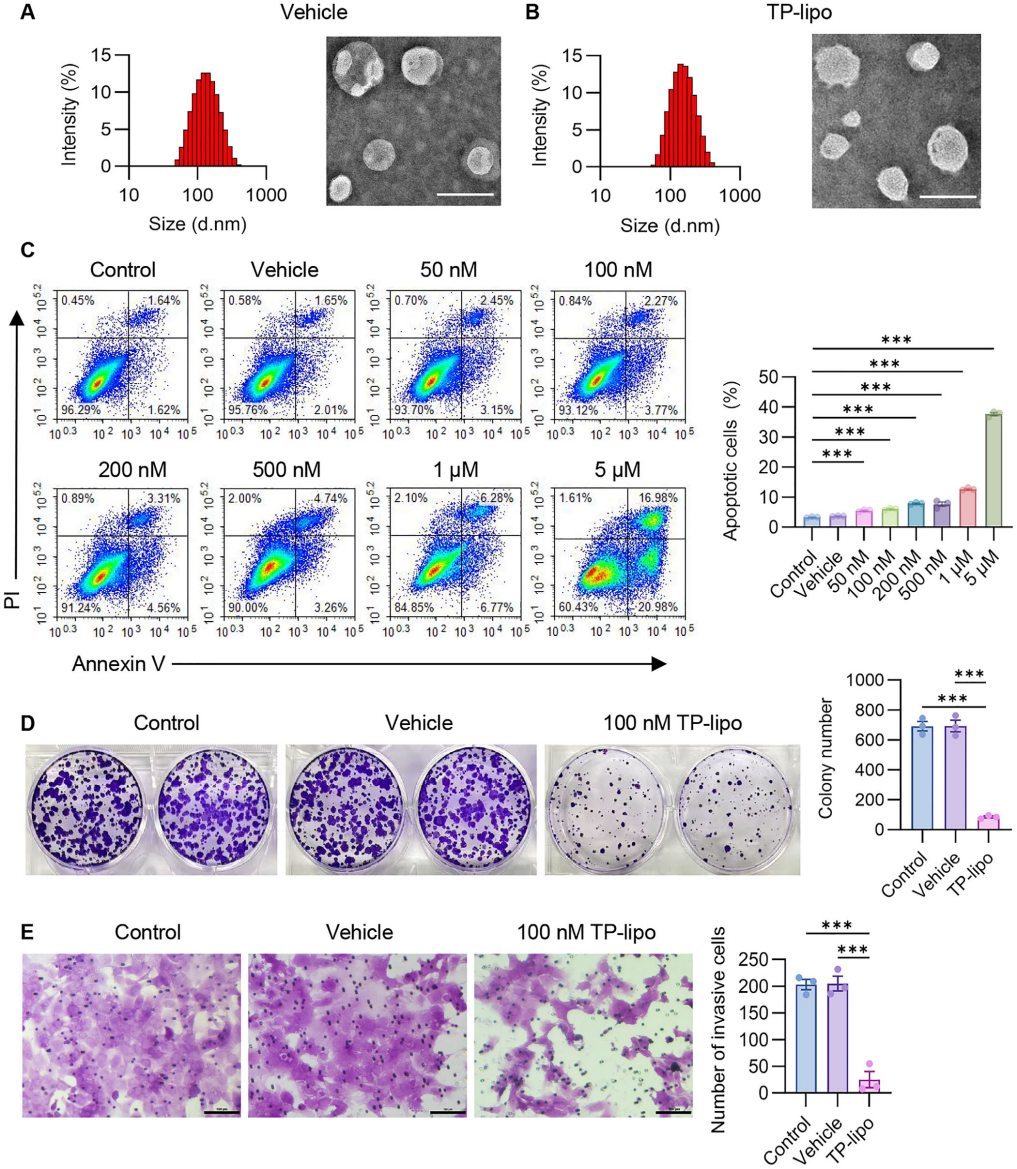
Characterization of tripterine liposomes (TP‐lipo) and its suppressive effects on KPC cells. (A) Hydrodynamic diameter of vehicle liposomes and TEM images. Scale bar = 50 nm. (B) Hydrodynamic diameter of TP‐lipo and TEM images. (C) Percentage of apoptotic KPC cells (Annexin V^+^/PI^+^ and Annexin V^+^/PI^−^) after treatment with different concentrations of TP‐lipo or vehicle liposomes. *n* = 3 per group. (D) Clonogenic capacity of KPC cells after treatment with 100 nM TP‐lipo or vehicle liposomes. *n* = 3 per group. (E) Invasion ability of KPC cells after treatment with 100 nM TP‐lipo or vehicle liposomes. Scale bar = 100 µm. *n* = 3 per group. The figure shows representative images; not all samples are shown. Data are presented as the mean ± standard error of the mean (SEM). ***p < 0.001.

To characterize the physicochemical properties of TP‐lipo, we evaluated the particle size, ζ potential, morphology of both TP‐lipo and vehicle liposomes, and encapsulation efficiency (EE%) of TP‐lipo. Dynamic light scattering (DLS) analysis showed that vehicle liposomes exhibited a mean hydrodynamic diameter of 109.53 ± 5.46 nm (Figure 1A), while the TP‐lipo displayed an average diameter of 134.10 ± 7.90 nm (Figure 1B). Transmission electron microscopy (TEM) images demonstrated that both TP‐lipo and vehicle liposomes exhibited a uniform spherical morphology (Figure 1A,B). The ζ potentials of TP‐lipo and vehicle liposomes were −45.7 ± 2.25 mV and −41.0 ± 1.87 mV, respectively. Furthermore, high‐performance liquid chromatography (HPLC) analysis revealed that TP‐lipo achieved an EE% of 87.71 ± 1.21%. Together, these data show that TP‐lipo exhibits a nanoscale size distribution, uniform spherical morphology, and high encapsulation efficiency.

### 2.2 TP‐lipo In Vitro Anticancer Activity

To evaluate the effects of TP‐lipo on pancreatic cancer cells, KPC cells were treated with TP‐lipo at concentrations varying from 50 nM to 5 µM, or vehicle liposomes for 24 h. Apoptosis was assessed using Annexin V/propidium iodide (PI) staining and flow cytometry. Compared with the control group, TP‐lipo significantly upregulated apoptosis in KPC cells at a concentration as low as 50 nM, with the apoptosis rate gradually increasing in a concentration‐dependent manner (Figure 1C). Additionally, the effect of TP‐lipo on the colony‐forming ability of KPC cells was examined using a colony formation assay. The results showed a clear decrease in the number of colonies in the TP‐lipo‐treated group compared with the control and vehicle liposome‐treated groups (Figure 1D). We further evaluated the effect of TP‐lipo on the invasion ability of KPC cells by a transwell assay. As shown in Figure 1E, a significantly smaller number of KPC cells penetrated the scaffold made of Matrigel in the TP‐lipo group compared to the control and vehicle groups, indicating that TP‐lipo suppressed KPC cells’ motility and invasion potential. Overall, our results suggest that TP‐lipo exhibits evident tumor‐suppressive effects in vitro, promoting apoptosis and inhibiting the clonogenicity and invasion of KPC cells.

### 2.3 TP‐lipo Inhibits Tumor Growth in Orthotopic PDAC Models

**FIGURE 2:**
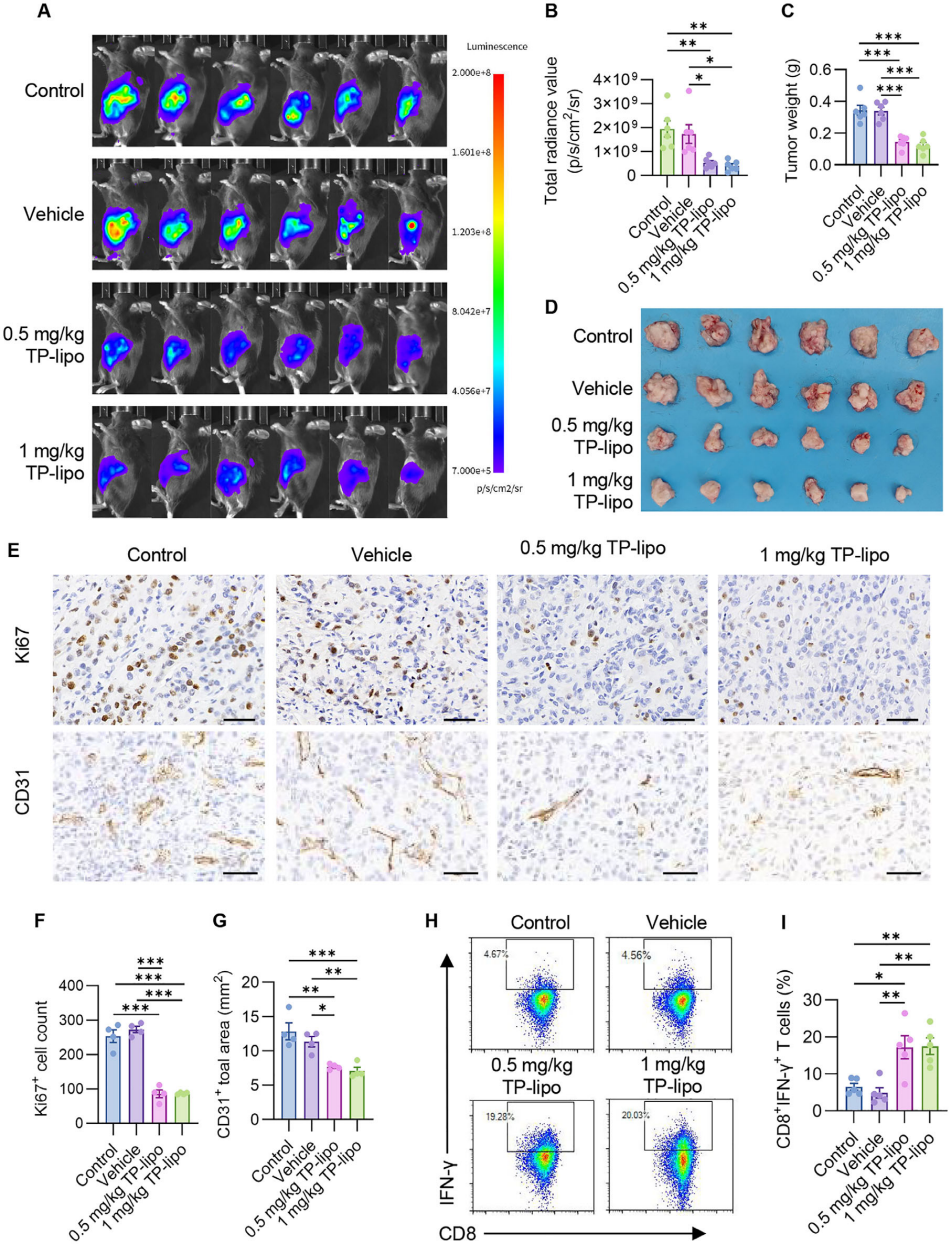
Inhibitory effects of tripterine liposomes (TP‐lipo) on mouse pancreatic ductal adenocarcinoma (PDAC) models. (A) In vivo bioluminescence imaging (BLI) images of mouse tumors on Day 8 after tumor implantation. *n* = 6 per group. (B) Statistical analysis of total radiation values from BLI images based on (A). (C) Statistical analysis of tumor weight in mice treated with control, vehicle liposomes, 0.5 mg/kg TP‐lipo, and 1 mg/kg TP‐lipo. *n* = 6 per group. (D) Gross morphology of tumor size after treatment with control, vehicle liposomes, 0.5 mg/kg TP‐lipo, or 1 mg/kg TP‐lipo. *n* = 6 per group. (E–G) Ki67^+^ cell count and CD31^+^ area in tumor tissues of mice treated with control, vehicle liposomes, 0.5 mg/kg TP‐lipo, and 1 mg/kg TP‐lipo. Scale bar = 50 µm. *n* = 4 per group. (H, I) Proportion of IFN‐γ‐secreting CD8^+^ T cells in peritoneal lavage fluid from control, vehicle liposome, 0.5 mg/kg TP‐lipo, and 1 mg/kg TP‐lipo groups. *n* = 5 per group. Data are presented as the mean ± standard error of the mean (SEM). *p < 0.05; **p < 0.01; ***p < 0.001.

To evaluate whether TP‐lipo inhibited tumor growth in vivo, we established orthotopic PDAC models using KPC‐luc cells. Tumor‐bearing mice were randomly allocated to four groups and received intraperitoneal injections with 5% dextrose solution (control group), vehicle liposomes (vehicle group), 0.5 mg/kg TP‐lipo, and 1 mg/kg TP‐lipo every 2 days. In vivo bioluminescence imaging (BLI) results revealed that both 0.5 mg/kg and 1 mg/kg TP‐lipo treatments significantly inhibited tumor growth compared with the vehicle and control groups, whereas no significant difference was observed between the vehicle and control groups (Figure 2A,B). Consistent with BLI findings, gross inspection revealed more extensive tumor growth and metastasis in the control and vehicle groups than in the TP‐lipo groups. Tumor burden, as measured by weight, was significantly lower in mice treated with either dose of TP‐lipo compared with the control and vehicle groups, with no significant difference between the vehicle and control groups (Figure 2C,D). Notably, no significant difference was detected between the two TP‐lipo doses, indicating that 0.5 mg/kg represents an effective dose (Figure 2A–D). Immunohistochemistry (IHC) staining of Ki67 and CD31 in tumor tissues demonstrated a marked reduction in Ki67^+^ tumor cells and CD31^+^ vascular area in the TP‐lipo groups compared with the control and vehicle groups, suggesting that TP‐lipo significantly suppresses the proliferation of tumor cells and the angiogenesis within the TME (Figure 2E–G). Flow cytometric analysis of peritoneal lavage fluid revealed a significant increase in interferon‐γ (IFN‐γ)‐secreting T cells in both 0.5 mg/kg and 1 mg/kg TP‐lipo groups (Figure 2H,I). Collectively, these results demonstrate that TP‐lipo has significant anti‐PDAC efficacy, characterized by reduced tumor cell proliferation, suppressed tumor angiogenesis, and increased IFN‐γ secretion by CD8^+^ T cells.

### 2.4 TP‐lipo Reprograms Tumor‐Associated Macrophages Toward an Antitumor Phenotype

**FIGURE 3:**
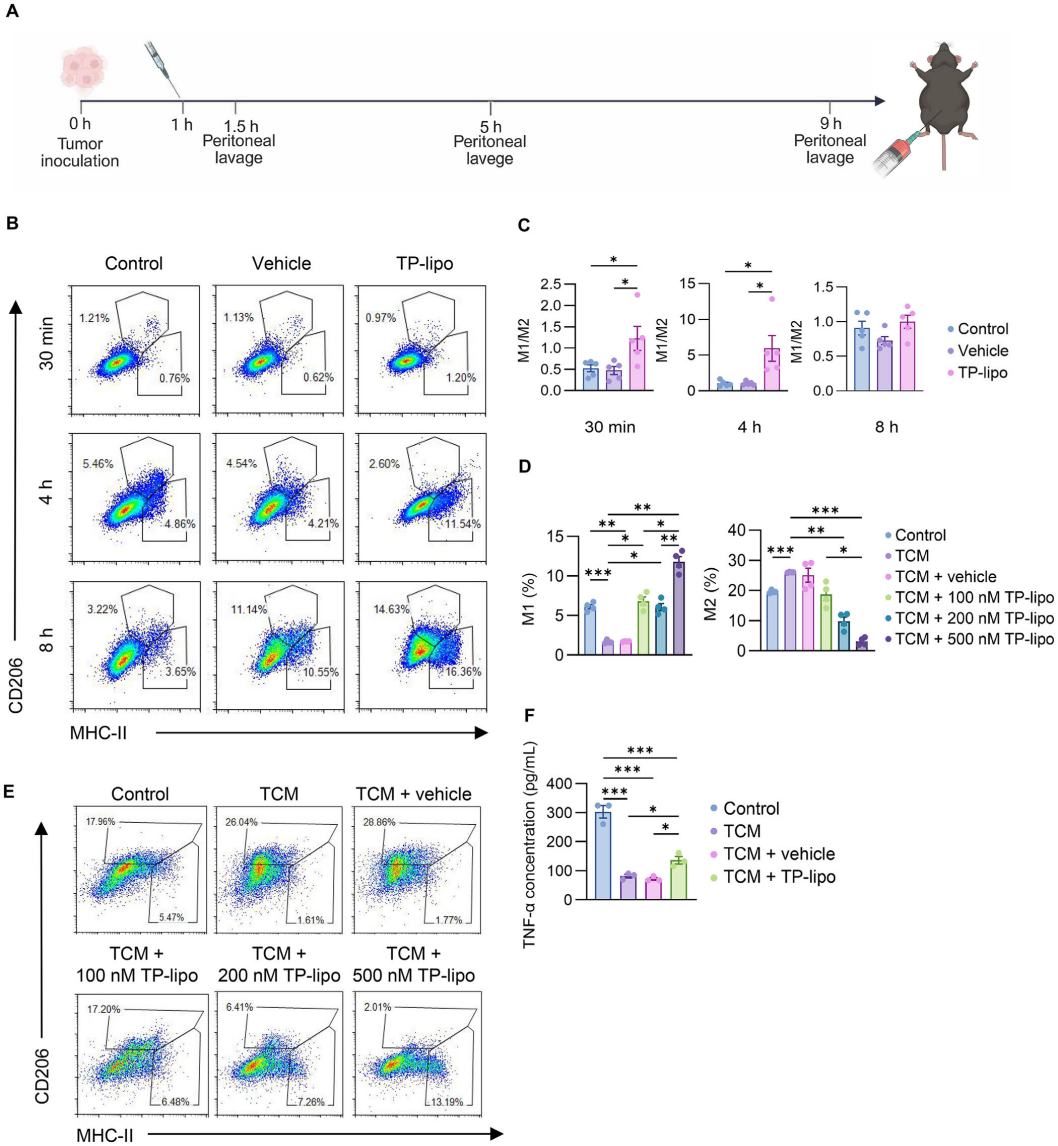
Effects of tripterine liposomes (TP‐lipo) on macrophage polarization. (A) Flowchart of multipoint intraperitoneal lavage fluid collection. Mice were intraperitoneally injected with KPC cells, then 1 h later with 5% dextrose, vehicle liposomes (0.5 mg/kg), or TP‐lipo (0.5 mg/kg). Peritoneal lavage fluid was collected at 30 min, 4 h, and 8 h post‐treatment for flow cytometry. (B, C) M1/M2 macrophage ratios in peritoneal lavage fluid at 30 min, 4 h, and 8 h post‑injection of TP‑lipo or vehicle liposomes. *n* = 5 per group. M1 and M2 macrophages were gated from the CD45^+^CD11b^+^F4/80^+^ population and identified as MHC‐II^+^CD206^−^ and CD206^+^MHC‐II^−^ cells, respectively. (D, E) Mouse bone marrow‑derived macrophages (BMDMs) were cultured for 48 h in tumor‑conditioned medium (TCM) supplemented with vehicle liposomes or TP‐lipo at concentrations of 100, 200, or 500 nM. Macrophage polarization was assessed by flow cytometry. *n* = 4 per group. (F) After 48 h of treatment with TCM and either vehicle liposomes or 500 nM TP‐lipo, Tumor necrosis factor‐α (TNF‐α) concentrations in macrophage supernatants were measured by ELISA. *n* = 3 per group. Data are presented as the mean ± standard error of the mean (SEM). *p < 0.05; **p < 0.01; ***p < 0.001.

Studies have reported that macrophage polarization toward the M2 phenotype occurs in the early stages of tumor development [19, 20]. During the precancerous stage of PDAC, macrophages are recruited to the periphery of the lesions [21]. At this stage, KRAS‐mutant tumor cells secrete factors such as IL‐4 and IL‐13, driving early infiltrating macrophages toward an M2 phenotype [21]. In turn, these macrophages secrete cytokines, including IL‐6, which further promote acinar‐to‐ductal metaplasia and the formation of PanIN lesions [21]. To explore the immediate effects of TP‐lipo on the TME shortly after tumor implantation, we analyzed the phenotype of macrophages in peritoneal lavage fluid at multiple time points following TP‐lipo or vehicle liposome injection. Mice were intraperitoneally inoculated with KPC cells. One hour later, mice intraperitoneally received a dose of 5% dextrose solution, 0.5 mg/kg vehicle liposomes, or 0.5 mg/kg TP‐lipo. Peritoneal lavage fluid was collected at 30 min, 4 h, and 8 h post‑treatment for flow cytometry. Macrophage polarization was evaluated using MHC‐II and CD206 as markers for M1‐ and M2‐like macrophages, respectively [22]. The gating strategy is shown in Figure S1. In both the control and vehicle groups, macrophages polarized toward the M2 phenotype after tumor inoculation. In contrast, TP‐lipo treatment promoted a shift toward the M1 phenotype in macrophages (Figure 3A). The M1/M2 ratios were significantly higher in the TP‐lipo treatment group than in the control and vehicle groups at 30 min. This difference further increased by 4 h but disappeared at 8 h, with the M1/M2 ratios among the three groups returning to comparable levels (Figure 3B,C).

Previous studies have reported that tumor cell‐conditioned medium (TCM) promotes macrophage polarization toward the M2 phenotype [23]. In the TME, such M2 polarization acts as a key driver for the progression of the immunosuppressive microenvironment in pancreatic cancer [24, 25]. To investigate whether TP‑lipo can reverse TCM‑induced M2 polarization, bone marrow–derived macrophages (BMDMs) were isolated and treated with TCM in the presence of either vehicle liposomes or varying concentrations of TP‑lipo for 48 h. The flow cytometry analysis revealed that TCM treatment increased the proportion of M2 macrophages, whereas TP‐lipo significantly inhibited this effect and promoted a concentration‐dependent shift toward the M1 phenotype (Figure 3D,E). Additionally, enzyme‐linked immunosorbent assay (ELISA) demonstrated that TCM treatment reduced TNF‐α secretion by macrophages. Compared to the vehicle group, TP‐lipo treatment markedly enhanced TNF‐α release (Figure 3F). As TNF‑α is a signature cytokine of M1‑polarized macrophages with direct tumor‑killing activity [26], its elevated level further supports the role of TP‑lipo in driving macrophage differentiation toward an anti‑tumor phenotype. These results suggest that TP‐lipo reprograms macrophage polarization from the M2 to the M1 phenotype.

### 2.5 TP‐lipo Enhances Effector T‐Cell Differentiation and Cytotoxic Function

**FIGURE 4:**
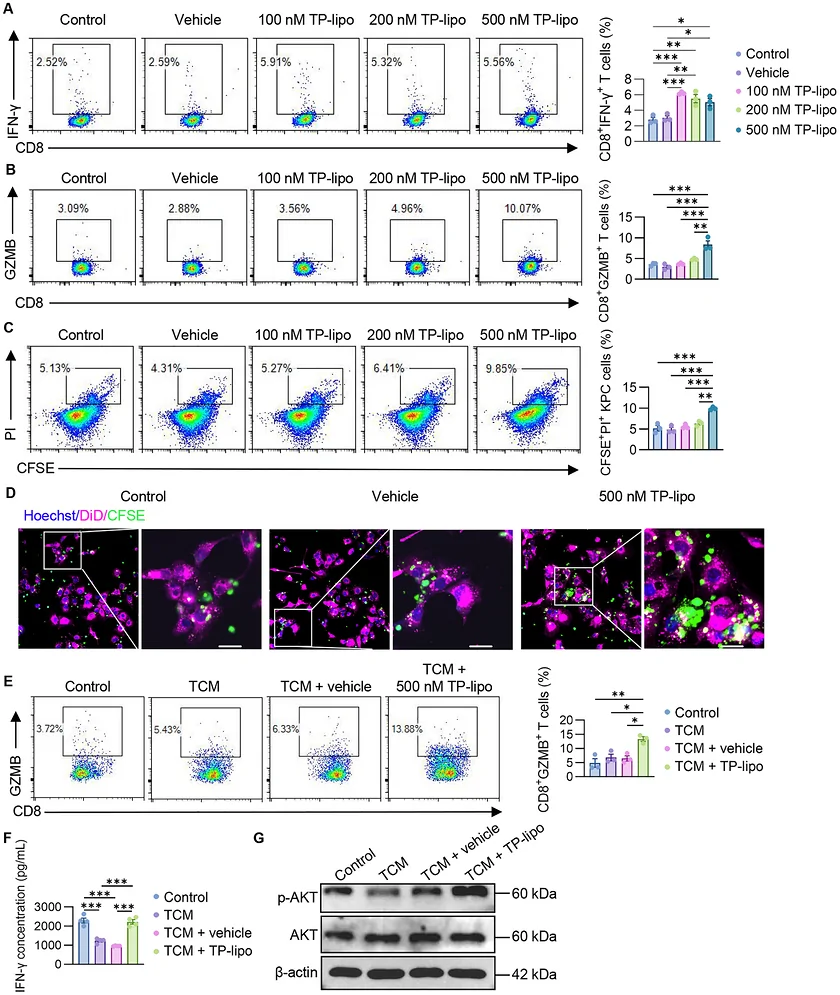
Effects of tripterine liposomes (TP‐lipo) on T‐cell differentiation and function. (A, B) After 24 h treatment with vehicle liposomes or TP‐lipo at different concentrations, the proportions of CD8^+^ interferon‐γ^+^ (IFN‐γ) T cells (A) and CD8^+^ granzyme B^+^ (GZMB^+^) T cells (B) were detected by flow cytometry. *n* = 3 per group. (C) Isolate mouse splenic T cells, pre‐treat with anti‐CD3 and anti‐CD28 antibodies, then treat with vehicle liposomes or different concentrations of TP‐lipo for 24 h. Co‐culture pre‐treated T cells with carboxyfluorescein diacetate succinimidyl ester (CFSE)‐labeled KPC cells for another 8 h, followed by propidium iodide (PI) staining and flow cytometric detection of dead KPC cells (CFSE^+^PI^+^). *n* = 3 per group. (D) Mouse splenic T cells were pretreated with anti‐CD3 and anti‐CD28 antibodies, treated with vehicle liposomes or TP‐lipo for 24 h, labeled with CFSE, and then co‐cultured with 1,1′‐dioctadecyl‐3,3,3′,3′‐tetramethylindodicarbocyanine, 4‐chlorobenzenesulfonate salt (DiD)‐labeled KPC cells for 8 h. Laser confocal imaging was used to observe T‐cell‐mediated lytic activity against KPC cells. Scale bar = 20 µm. (E) Bone marrow–derived macrophages (BMDMs) treated with tumor‐conditioned medium (TCM) and either vehicle liposomes or 500 nM TP‐lipo were co‐cultured with anti‐CD3 and anti‐CD28 antibody‐pretreated T cells for 24 h. The proportion of CD8^+^GZMB^+^ T cells was detected by flow cytometry. *n* = 3 per group. (F) IFN‐γ concentrations in the co‐culture supernatant were measured by ELISA. *n* = 4 per group. (G) p‐AKT and total AKT expression in T cells were assessed by Western blot. Data are presented as the mean ± standard error of the mean (SEM). *p < 0.05; **p < 0.01; ***p < 0.001.

To investigate whether TP‑lipo directly promotes cytotoxic T‑cell differentiation, T cells treated with TP‑lipo or vehicle liposomes for 24 h were analyzed by flow cytometry. Results showed that treatment with 100 nM TP‐lipo markedly elevated the proportion of IFN‐γ^+^ cytotoxic T cells compared with the vehicle group, but no further enhancement was observed at higher concentrations (Figure 4A). In contrast, a significant increase in granzyme B^+^ (GZMB) cytotoxic T cells was observed only in the 500 nM TP‑lipo group (Figure 4B). Based on these results, we next co‐cultured TP‑lipo‑treated T cells with carboxyfluorescein diacetate succinimidyl ester (CFSE)‐labeled KPC tumor cells to evaluate their cytotoxic activity. Flow cytometry assessment showed that TP‐lipo augmented T cell‐mediated cytotoxicity against KPC cells in a concentration‐dependent fashion, with 500 nM TP‑lipo producing the most pronounced increase in tumor cell death, identified as CFSE^+^PI^+^ (Figure 4C). Confocal imaging also revealed that TP‐lipo‐treated T cells (CFSE‐labeled) exhibited enhanced ability to infiltrate and kill 1,1'‐dioctadecyl‐3,3,3',3'‐tetramethylindodicarbocyanine, 4‐chlorobenzenesulfonate salt (DiD)‐labeled KPC cells compared with the control and vehicle groups, where only minimal cytotoxicity was observed (Figure 4D).

We next explored whether macrophages exposed to TCM and TP‑lipo could modulate the differentiation of cytotoxic T cells. BMDMs were first pretreated for 48 h with TCM, TCM + vehicle, or TCM + 500 nM TP‑lipo, and then co‑cultured with T cells for 24 h. Flow cytometry results showed that macrophages pretreated with TCM + TP‑lipo promoted the differentiation of GZMB‑secreting cytotoxic T cells (Figure 4E) compared with other groups. Correspondingly, ELISA of the co‑culture supernatant revealed that TCM treatment reduced IFN‑γ levels, whereas TP‑lipo restored IFN‑γ secretion in T cells (Figure 4F). Western blot analysis of T‑cell lysates revealed that phosphorylation of AKT (p‑AKT), a central mediator of cytotoxic signaling, was suppressed in the TCM and TCM + vehicle groups. TP‑lipo intervention reversed this suppression, resulting in higher p‑AKT levels than those in the TCM and TCM + vehicle groups (Figure 4G). Together, these findings indicate that TP‐lipo enhances cytotoxic effector T‑cell differentiation through both direct and macrophage‑mediated pathways.

### 2.6 TP‐lipo Synergizes With PD‐1 Blockade to Improve Antitumor Efficacy

**FIGURE 5:**
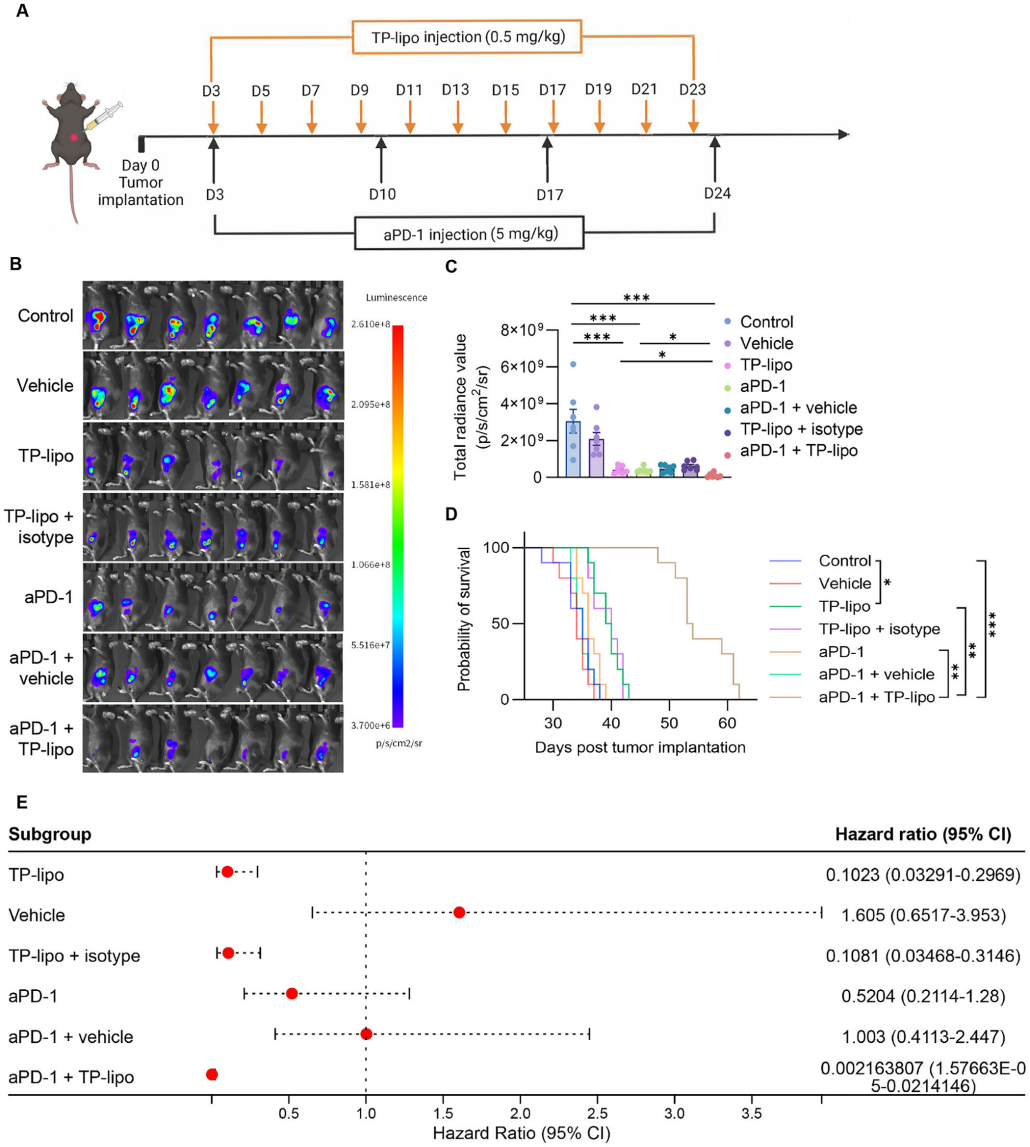
The effect of tripterine liposomes (TP‐lipo) in combination with PD‐1 antibody (aPD‐1) on tumor burden and survival time in mice. (A) Schematic of the treatment regimen with TP‐lipo and aPD‐1 in the pancreatic ductal adenocarcinoma (PDAC) mouse model. (B, C) In vivo bioluminescence imaging (BLI) results at Day 18 post‐tumor implantation (B), and statistical plot (C) of tumor‐bearing mice treated with vehicle liposomes, TP‐lipo, and/or aPD‐1. *n* = 7 per group. Data are presented as the mean ± standard error of the mean (SEM). *p < 0.05; ***p < 0.001. (D) Survival curves were compared using the log‑rank test with Bonferroni correction for multiple comparisons. A corrected *p* value < 0.05 was considered statistically significant. *n* = 10 per group. (E) Forest plot of Cox proportional hazards regression analysis. Hazard ratios (HRs) with 95% confidence intervals (CIs) are shown. *n* = 10 per group.

Given the significant antitumor activity of TP‐lipo, we further explored its synergistic effects with aPD‐1 using an intraperitoneal PDAC model. Treatment was initiated on Day 3 post‐tumor implantation. Mice received TP‐lipo every other day for a total of 11 doses, and aPD‐1 weekly for a total of 4 doses (Figure 5A). BLI results revealed that monotherapy with either TP‐lipo or aPD‐1 significantly suppressed tumor growth compared to the vehicle and control groups. Notably, the combination group exhibited the lowest bioluminescence radiance values, with a significant difference in tumor burden compared with the aPD‐1 and TP‐lipo monotherapy groups (Figure 5B,C). Survival analysis revealed that aPD‐1 monotherapy did not prolong median survival, whereas TP‐lipo monotherapy did. Importantly, the median survival in the combination group was significantly higher than that in the aPD‐1 and TP‐lipo monotherapy groups (Figure 5D), demonstrating the substantial clinical potential of combining TP‐lipo with aPD‐1. Cox proportional hazards regression analysis showed that TP‐lipo monotherapy and combination treatment with aPD‐1 and TP‐lipo were significantly associated with improved overall survival in mice, whereas aPD‐1 monotherapy was not. Notably, the combination group exhibited the lowest hazard ratio (HR) among all the treatment groups, indicating the greatest survival benefit (Figure 5E). Taken together, these results indicate that TP‐lipo prolongs survival in mouse PDAC models and improves the therapeutic efficacy of PD‐1 blockade in combination treatment.

### 2.7 Biosafety Evaluation

**FIGURE 6:**
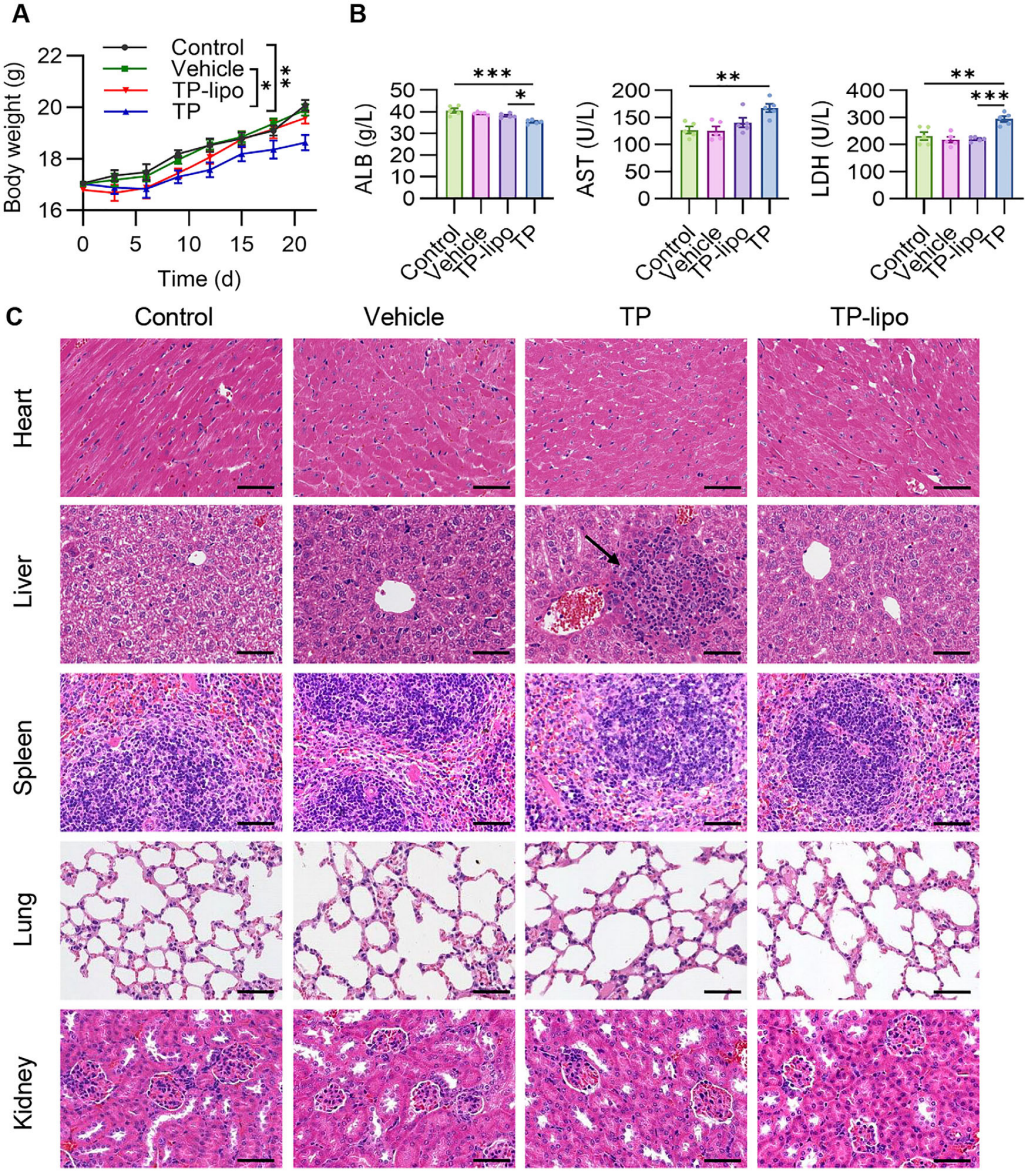
Blood biochemistry and H&E staining of mouse organs after tripterine (TP) and tripterine liposomes (TP‐lipo) administrations. (A) Changes in mouse body weight following administration of TP or TP‐lipo. *n* = 8 per group. (B) Abnormal indicators in serum biochemical analyses following 11 injections of vehicle liposomes, TP, or TP‐lipo. ALB, Albumin; AST, aspartate aminotransferase; LDH, lactate dehydrogenase. *n* = 5 per group. (C) Histopathological changes in major organs after 11 injections of vehicle liposomes, TP, and TP‐lipo. The arrow indicates the locations of pathological changes. Scale bar = 50 µm. Data are presented as the mean ± standard error of the mean (SEM). *p < 0.05; **p < 0.01; ***p < 0.001.

To evaluate whether liposome encapsulation can reduce TP toxicity, healthy C57BL/6N mice received 11 doses of vehicle liposomes, TP, or TP‐lipo. During the treatment period, body weight progressively increased in the control and vehicle groups. Although mice treated with TP also gained weight, the increase was significantly smaller than that of the control and vehicle groups. By contrast, body weight gain in the TP‐lipo group was not significantly different from that in the control or vehicle groups (Figure 6A). To further evaluate the in vivo toxicity profile of the treatments, serum biochemical parameters were analyzed, and major organs were subjected to hematoxylin and eosin (H&E) staining. Serum biochemical analysis revealed that TP administration significantly increased aspartate aminotransferase (AST) and decreased albumin (ALB) levels, indicating hepatocellular injury and impaired liver function. In contrast, no significant alterations were observed in the TP‐lipo group, suggesting reduced hepatotoxicity following liposomal encapsulation. Serum lactate dehydrogenase (LDH), a non‐specific marker of tissue injury, was markedly elevated in the TP group but not in the TP‐lipo group (Figure 6B). Consistently, histological analysis revealed marked perivascular inflammatory cell infiltration in the liver tissue of TP‐treated mice, accompanied by focal hepatocellular damage. In contrast, these alterations were not observed in the TP‐lipo group (Figure 6C). Overall, these findings suggest that liposomal encapsulation alleviates the in vivo toxicity associated with TP.

## 3 Discussion

The high lethality of PDAC is the overall outcome of its clinical characteristics—late diagnosis, coupled with its aggressive cell‐autonomous biology and profoundly immunosuppressive TME. Approximately 60% of patients are diagnosed with metastasis [27]. Additionally, most PDAC patients undergo cachexia due to impaired pancreatic function, cancer‐associated metabolic and systemic disruptions, and dysregulated gastrointestinal activity, resulting in up to 60% of patients not being suitable for subsequent treatments [27]. The highly fibrotic, strongly immunosuppressive TME of PDAC also impairs drug delivery and immunotherapy efficacy. Furthermore, intratumoral heterogeneity further complicates therapeutic intervention [27]. Therefore, identifying agents that simultaneously suppress tumor growth and modulate the immunosuppressive TME is urgently needed. The present study has validated that TP‐lipo suppresses PDAC tumor growth and reshapes the TME immune landscape in vivo. It directly inhibits KPC cells by suppressing colony formation and invasion, as well as promoting apoptosis. Furthermore, TP‐lipo drives macrophage polarization toward an antitumor phenotype and enhances cytotoxic T‐cell activation in ascites. Taken together, TP‐lipo exerts anti‐PDAC activity through a synergistic mechanism that directly inhibits tumor growth and reverses the immunosuppressive TME, thereby offering a promising strategy to overcome current therapeutic limitations in PDAC.

Although the antitumor property of TP has long been recognized, its clinical application is limited by poor solubility, low bioavailability, and systemic toxicity [28]. Liposomal encapsulation is a safe nanoscale strategy that increases solubility and intestinal uptake while minimizing premature drug release and off‑target exposure, thereby reducing toxicity [29]. Consistent with this, our study confirms the attenuated acute toxicity of TP‑lipo in hepatic tissue. Previous studies demonstrated that TP induced pancreatic cancer cell death by inhibiting OGT‐mediated O‐GlcNAcylation of Sp1, thereby blocking Sp1 nuclear translocation and suppressing NF‐κB and HSF1 activity, resulting in down‐regulation of pro‐survival proteins such as HSP70 [30]. Consistent with these findings, our results showed that TP‐lipo promoted KPC cell apoptosis in a dose‐dependent manner, reaffirming its cytotoxic effect on PDAC tumor cells. Furthermore, TP‐lipo was observed to inhibit the invasive capacity and clonogenic potential of KPC cells, corroborating its direct antitumor activity.

Macrophage polarization is a dynamic process by which these innate immune cells adopt distinct functional phenotypes in response to microenvironmental signals [31]. Classically activated M1 macrophages exert potent antitumor functions by producing NO, reactive oxygen species (ROS), and pro‐inflammatory cytokines such as TNF‐α and IL‐1β [32]. These mediators not only directly kill tumor cells but also activate Th1 and cytotoxic T lymphocytes, thereby linking innate and adaptive antitumor immunity [32]. In contrast, alternatively activated M2 macrophages are characterized by the expression of Arg‐1, CD206, and anti‐inflammatory cytokines such as IL‐10 and TGF‐β [33]. While M2 macrophages play essential roles in tissue repair and immune regulation, within the TME, they frequently promote tumor progression by facilitating angiogenesis, immune suppression, and metastasis [34]. Importantly, the TME can reprogram M1 macrophages toward an M2‐like phenotype through metabolic stress, cytokine signaling, and immune checkpoint pathways such as CD47‐signal regulatory protein α [35]. In the present study, we cultured macrophages with TCM to mimic the in vivo influence of tumor cells on macrophage polarization. Our results showed that, compared to standard culture conditions, TCM increased the proportion of M2 macrophages. In contrast, TP‑lipo treatment dose‑dependently reduced the M2 subset and increased the proportion of M1 macrophages, suggesting that TP‐lipo is capable of reprogramming macrophage polarization toward an antitumor phenotype. Consistent with our findings, previous studies have shown that TP suppresses STAT6 phosphorylation and restrains M2‐like polarization of tumor‐associated macrophages, thereby alleviating tumor‐associated immunosuppression [36]. TP has also been implicated in the regulation of macrophage polarity through reducing the secretion of lactic acid from tumor cells, thereby depriving the fuel required for M2 macrophage polarization [37]. Although the precise mechanisms underlying TP‐lipo‐induced macrophage reprogramming remain to be fully elucidated, many studies on TP suggest its role in promoting macrophage polarization toward a more immunostimulatory phenotype [12, 36, 38]. Macrophages express phosphatidylserine (PS)‐recognition receptors such as Tim‐4 and MerTK [39], while PS itself is a well‐established phagocytic “eat‐me” signal [40]. In line with this, previous studies have shown that PS‐modified liposomes enhance uptake by macrophages, and 1,2‐dioleoyl‐sn‐glycero‐3‐phospho‐L‐serine (DOPS)‐modified liposomes showed the highest uptake in RAW264.7 macrophages among other PS‐based formulations [41]. DOPS‐based lipid nanoparticles were also reported to be preferentially taken up by alveolar macrophages in vivo in pulmonary fibrosis models [42]. Together, these findings suggest that incorporating DOPS may facilitate TP‐lipo interaction with macrophages. Overall, these findings suggest that TP‐lipo may exert immunomodulatory effects, at least in part, by reprogramming macrophages.

The ability of TP‐lipo to enhance cytotoxic T‐cell responses may represent an important mechanism underlying its antitumor activity in PDAC. Wang et al. [43] reported that endoplasmic reticulum (ER)‐targeting TP nanoparticles induced pronounced ER stress, leading to exposure of calreticulin and release of high‐mobility group box 1 protein (HMGB1) and ATP. These damage‐associated molecular signals further contributed to the recruitment and maturation of dendritic cells, thereby enhancing the expansion of cytotoxic T cells. In addition, Kong et al. [44] demonstrated that TP interacts with Src homology 2 domain‐containing protein tyrosine phosphatase‐2 to activate the JAK/STAT1 pathway, resulting in upregulation of MHC‐I in B16F10 melanoma cells and, consequently, stronger cytotoxic T‐cell responses in vivo. Consistent with these findings, TP‐lipo treatment directly increased the percentage of IFN‐γ^+^ and GZMB^+^ cytotoxic T cells, suggesting that TP‐lipo may enhance antitumor immunity by promoting cytotoxic T‐cell responses. In addition, macrophages also play a key role in regulating T‐cell differentiation in the TME. Our co‐culture results demonstrated that TP‐lipo‐pretreated macrophages significantly increased the secretion of GZMB and IFN‐γ in cytotoxic T cells. Meanwhile, the upregulation of p‐AKT expression levels in T cells further corroborates the activation of the effector T‐cell differentiation pathway. Prior studies have reported that AKT signaling participates in CD8^+^ T‐cell effector differentiation by integrating TCR/co‐stimulatory and cytokine‐derived cues, and the activation of AKT further triggers mTOR‐ and Foxo1‐related downstream pathways implicated in the balance between effector and memory differentiation [45, 46]. Together, these findings suggest that TP‐lipo may enhance antitumor immunity in PDAC by promoting cytotoxic T‐cell differentiation and activation, potentially through both direct effects and macrophage‐mediated mechanisms.

The synergistic antitumor effect observed with TP‐lipo and PD‐1 blockade suggests that TP‐lipo may sensitize PDAC to immune checkpoint inhibition, thereby suppressing tumor progression and prolonging survival. PD‐1 and its ligand PD‐L1 constitute a key immune checkpoint pathway, and their interaction dampens adaptive immunity by suppressing effector T cells and promoting regulatory T‐cell‐mediated immunosuppression, thereby preserving immune homeostasis [47]. PDAC cells frequently exhibit elevated PD‐L1 expression, which contributes to immune evasion by impairing T‐cell‐mediated antitumor responses [48]. The enhanced efficacy of TP‐lipo combined with PD‐1 blockade may involve modulation of the PD‐1/PD‐L1 axis. Previous studies have reported that TP can upregulate PD‐L1 expression in KPC cells, suggesting that pancreatic tumor cells may develop an adaptive PD‐L1 response to TP‐induced stress [49]. Besides, TP has been found to inhibit the expression of PD‐L1 on dendritic cells [50]. Accordingly, PD‐1 blockade may help overcome adaptive immune evasion associated with TP‐lipo treatment, thereby enhancing antitumor immunity.

PDAC is characterized by a dense desmoplastic stroma that severely restricts the intratumoral delivery and penetration of nanotherapeutics. Although nanoparticles in the 50–200 nm size range may accumulate in tumors after vascular extravasation, effective penetration into the dense tumor parenchyma remains a major challenge [51]. Despite this barrier, TP‐lipo significantly suppressed PDAC growth and prolonged survival in PDAC‐bearing mice. Accumulating evidence has implicated M2 macrophages in stromal deposition and immunosuppressive remodeling of the PDAC TME [52, 53]. Consistent with this notion, TP‐lipo promoted macrophage reprogramming from an M2 to an M1 phenotype in ascites from the intraperitoneal PDAC model, suggesting that TP‐lipo might alleviate the stromal and immunosuppressive features of the PDAC microenvironment. The relative contributions of immune microenvironment modulation and direct tumor cell suppression to the antitumor activity of TP‐lipo in PDAC remain to be elucidated.

Despite the promising antitumor efficacy of TP‐lipo observed in this study, several questions remain to be addressed. The molecular mechanisms underlying its regulation of macrophage polarization and T‐cell responses remain to be further elucidated. Moreover, TP‐lipo was administered exclusively by intraperitoneal injection in the present study; thus, future studies evaluating clinically relevant delivery routes will be important for more fully defining its translational potential.

In conclusion, TP‐lipo demonstrated potent antitumor activity and favorable tolerability in preclinical PDAC models. Its therapeutic efficacy may derive from both direct tumor suppression and remodeling of the immune microenvironment, accompanied by macrophage reprogramming and enhanced cytotoxic T‐cell responses. Furthermore, its ability to potentiate PD‐1 blockade highlights TP‐lipo as a promising combinatorial strategy for PDAC treatment.

## 4 Materials and Methods

### 4.1 Animals

Female C57BL/6N mice (6–8 weeks old) were obtained from Vital River (Beijing, China). All animal experiments were conducted in accordance with protocols approved by the Animal Care and Use Committee of Sichuan University.

### 4.2 Cell Culture and Conditioned Medium Preparation

The murine KPC pancreatic cancer cell line was purchased from Shanghai Model Organisms Center, Inc. Cells were cultured in RPMI‐1640 medium (Gibco, USA) supplemented with 10% fetal bovine serum (FBS, Gibco, USA), 100 U/mL penicillin, 100 µg/mL streptomycin (Gibco, USA), 1 × MEM NEAA (Gibco, USA), and 1 × sodium pyruvate (Sigma, USA) at 37°C in a humidified incubator containing 5% CO_2_. When the confluence of KPC cells reached 80%–90%, cells were harvested for implantation, and the culture supernatant was collected and centrifuged at 2000 rpm for 5 min to remove cellular debris. The resulting TCM was collected and stored at −80°C until use.

### 4.3 Preparation of TP Liposomes and Vehicle Liposomes

Hydrogenated soy phosphatidylcholine (HSPC) and DOPS were purchased from Avanti (USA), and cholesterol was obtained from AVT Pharmaceutical Tech Co. (Shanghai). In this study, liposomes were formulated at a molar ratio of HSPC: cholesterol: DOPS = 4.8: 1.8: 3.4. The TP‐to‐total lipid ratio used during TP‐lipo synthesis was 1 mg/10 mg. Briefly, dissolve the HSPC, cholesterol, DOPS, and TP in chloroform, then remove the chloroform by vacuum drying to obtain a uniform orange‐yellow film. Subsequently, add 10% sucrose aqueous solution for thorough hydration. The resulting orange‐yellow liposome solution is filtered through a 0.22 µm PES membrane to obtain TP‐lipo. A similar approach is utilized for the preparation of vehicle liposomes. The particle size and ζ potential of TP‐lipo and vehicle liposomes were determined using Zetasizer (Malvern, UK). The morphology of liposomes was examined by TEM (JEM‐2100Plus, JEOL, Japan).

### 4.4 Endotoxin Assessment of Liposomes

The endotoxin levels in TP‐lipo and vehicle liposomes were measured using a Tachypleus Amebocyte Lysate Kit (Xiamen Bioendo Technology Co., Xiamen, China) according to the manufacturer's instructions. Only liposomes with endotoxin levels below 0.25 EU/mL were used in subsequent experiments.

### 4.5 Transwell Invasion Assay

For the invasion assay, Matrigel (BD Biosciences, USA) was diluted 1:6 in serum‐free RPMI‐1640 medium and used to coat the upper chamber of the transwell inserts (50 µL per well). After hydration with 100 µL of 1% bovine serum albumin (BSA), 1×10^5^ KPC cells suspended in serum‐free RPMI‐1640 medium were seeded into the upper chamber. The lower chamber was filled with 600 µL RPMI‐1640 medium containing 10% FBS. KPC cells were treated with vehicle liposomes or 100 nM TP‐lipo and incubated for 24 h. After incubation, non‐invading cells on the upper surface of the membrane were gently removed with a cotton swab. Cells that had invaded through the Matrigel‐coated membrane were fixed with 4% paraformaldehyde for 10 min, stained with 0.5% crystal violet, and quantified by ImageJ software (NIH, USA).

### 4.6 Colony Formation Assay

KPC cells were seeded into 6‐well plates at a density of 500 cells per well and treated with 100 nM TP‐lipo or vehicle liposomes. After 12 days of routine culture, cells were washed twice with PBS and fixed with 4% paraformaldehyde for 15 min at room temperature. Subsequently, cells were stained with 0.5% crystal violet solution for 30 min. Following staining, colonies were visualized and counted under a microscope, with a colony defined as a cluster of ≥ 50 cells.

### 4.7 Apoptosis Analysis by Flow Cytometry

KPC cells were treated with TP‐lipo (50 nM to 5 µM) or vehicle liposomes for 24 h. Cells were harvested and washed twice with phosphate‐buffered saline (PBS). The cell pellets were resuspended in 100 µL of 1× binding buffer and stained with 5 µL Annexin V‐FITC and PI for 10 min at room temperature in the dark (FITC Annexin V Apoptosis Detection Kit, BD, USA). Following incubation, 400 µL of 1 × binding buffer was added to terminate staining, and apoptotic cells were analyzed by flow cytometry using a NovoCyte Advanteon (Agilent, USA).

### 4.8 PDAC Mouse Models and Treatments

KPC cells stably expressing firefly luciferase (KPC‐luc) were generated by lentiviral infection and puromycin screening. To evaluate the in vivo antitumor activity of TP‐lipo, an orthotopic PDAC mouse model was established to recapitulate the natural progression of the disease. Briefly, mice were anesthetized and subjected to laparotomy. The pancreas was gently exteriorized, and 25 µL of tumor cell suspension (1 × 10^7^ cells/mL) was injected into the pancreatic parenchyma using an insulin syringe. The appearance of clear bubbles on the pancreatic surface indicated successful implantation. The abdominal wall and skin were closed with a double‐layer suture, and mice were placed on a heating pad for recovery. Then, mice were randomly allocated to four groups: control (5% dextrose solution), vehicle, 0.5 mg/kg TP‐lipo, and 1 mg/kg TP‐lipo. Treatments were initiated on Day 3 post‐implantation and administered every other day. Tumor progression was monitored by BLI. Mice were intraperitoneally injected with D‐luciferin (150 mg/kg; IVISbrite D‐Luciferin Potassium, Revvity, USA), and imaging was carried out 10–15 min post‐injection using an IVIS imaging system.

An intraperitoneal PDAC model was established to evaluate the therapeutic efficacy of TP‐lipo in combination with aPD‐1 (InVivoMAB, USA, CAT#BE0146). The model was constructed by intraperitoneal injection of 100 µL KPC‐luc cell suspension (2.5 × 10^6^ cells/mL). Tumor‐bearing mice were then randomly assigned to receive treatments in the following groups: control (5% dextrose solution), vehicle, TP‐lipo, TP‐lipo + isotype, aPD‐1, aPD‐1 + vehicle, or aPD‐1 + TP‐lipo. Starting on Day 3 post‐inoculation, mice received either aPD‐1 (5 mg/kg, administered weekly for a total of 4 doses, InVivoMAb) and/or TP‐lipo (0.5 mg/kg, administered every other day for a total of 11 doses). Tumor progression was monitored via in vivo BLI, and long‐term survival was systematically tracked.

### 4.9 Isolation and Treatment of BMDMs

Bone marrow cells were isolated from the tibiae and femurs of C57BL/6N mice. Briefly, following euthanasia by cervical dislocation, mice were immersed in 75% ethanol for 5 min for surface sterilization. Subsequently, the tibiae and femurs were carefully dissected and bone marrow cells were harvested by flushing the bone cavities with sterile PBS. Then, the collected cells were treated with red blood cell lysis buffer for 3 min, washed with PBS and cultured in RPMI‐1640 medium (Gibco, USA) supplemented with 10% FBS (Gibco, USA) and 20 ng/mL recombinant murine macrophage colony‐stimulating factor (M‐CSF, Novoprotein, China). After 5 days of culture, BMDMs were obtained and subjected to treatment with TCM in the presence of 100, 200, 500 nM TP‐lipo or vehicle liposomes. M1 or M2 polarization groups were established by co‐treating with 50 ng/mL LPS (Medchemexpress, USA) and 10 ng/mL IFN‐γ (Novoprotein, China) or by treating with 20 ng/mL IL‐4 (Novoprotein, China), respectively. These two groups served as a reference during subsequent flow cytometry gating. Following 48 h of treatment, BMDMs were harvested for flow cytometric analysis to determine the M1 and M2 macrophage subsets. The gating strategy for macrophages is shown in Figure S1. Antibodies used for staining are documented in Table S1. Cell‐cultured supernatants were collected, and TNF‐α levels were measured by ELISA (mouse TNF‐α uncoated ELISA kit, Invitrogen, USA).

### 4.10 In Vitro T‐Cell Activation and Cytotoxicity Analysis

Splenic lymphocytes were isolated from C57BL/6N mice using a density gradient centrifugation method at 800 × g for 30 min using lymphocyte isolation solution (Dakewe, China). The lymphocytes were collected and resuspended in complete RPMI‐1640 medium (Gibco, USA) supplemented with 15 ng/mL IL‐2 (Novoprotein, China), 0.5 µg/mL anti‐CD28 (BioLegend, USA) and 0.02 µg/mL anti‐CD3 antibodies (BioLegend, USA). After 24 h of culture, the medium was replaced with different formulations. For the control, cells were cultured in RPMI‐1640 containing 15 ng/mL IL‐2 alone. For the TCM group, cells were cultured in a 1:1 mixture of RPMI‐1640 (with 15 ng/mL IL‐2) and TCM. For the treatment groups, the same 1:1 mixture was supplemented with 100, 200, or 500 nM TP‐lipo, or with vehicle liposomes. Following an additional 24 h of incubation, T cells were harvested for flow cytometric analysis. Antibodies used for staining are documented in Table S1.

For cytotoxicity assessment, KPC cells were labeled with CellTrace CFSE (Invitrogen, USA) according to the manufacturer's instructions. Labeled KPC cells were then co‐cultured with treated T cells for 8 h. After PI staining, the proportion of dead KPC cells (CFSE^+^PI^+^) was determined by flow cytometry.

To further visually assess the cytotoxic activity of T cells against KPC cells, T cells pretreated with TCM and TP‐lipo were labeled with CFSE, while KPC cells were stained with DiD (Beyotime, China). The two cell populations were then co‑incubated for 8 h. Following co‑culture, cells were fixed, stained with Hoechst (Beyotime, China), and imaged using a confocal microscope (SpinSR10, Olympus, Japan).

### 4.11 Co‐Culture of T Cells With BMDMs

BMDMs were pretreated for 48 h with TCM, TCM + vehicle, or TCM + 500 nM TP‑lipo. BMDMs were then collected and added to activated T cells pre‐treated with IL‐2, anti‐CD28, and anti‐CD3 antibodies. After 24 h of co‐culture, T cells were harvested for flow cytometry analysis, and culture supernatants were collected for the measurement of IFN‑γ levels using a mouse IFN‑γ uncoated ELISA kit (Invitrogen, USA). Antibodies used for staining are documented in Table S1. Western blot was used to detect the expression levels of p‐AKT (S473) (1:1000, Cell Signalling, USA) and pan‐AKT (1:500, Abcam, UK) in T cells from different groups to elucidate the activation status of core signaling pathways driving T‐cell killing capacity.

### 4.12 Histopathology

The hearts, livers, spleens, lungs, and kidneys were collected from mice. The organs were fixed in 4% paraformaldehyde for 24 h and embedded in paraffin. Paraffin blocks containing the organs were sectioned into 4‐µm‐thick slices and stained with hematoxylin and eosin. The stained sections were scanned using a Pannoramic MIDI scanner (3DHISTECH Ltd., Hungary).

### 4.13 Immunohistochemical Staining

Orthotopic PDAC tumors were obtained and fixed in 4% paraformaldehyde for 24 h. Then tumor tissues were embedded in paraffin and cut into 4‐micrometer‐thick slices. The slices were stained for Ki67 (1:200, AB16667, Abcam, UK) and CD31 (1:500, GB113151, Servicebio, China) to visualize the proliferation and the angiogenesis, respectively. An HRP‑conjugated goat anti‑rabbit IgG (1:500, GB23303, Servicebio, China) was used as the secondary antibody for detection. The number of Ki67^+^ cells and CD31^+^ area in tissue sections were quantified by ImageJ. CD31^+^ area was quantified from 10 × fields, whereas Ki67^+^ cells were quantified from 40 × fields.

### 4.14 In Vivo Toxicity Evaluation

The toxicity of TP‐lipo and free TP was evaluated in C57BL/6N mice. Mice were randomly divided into four groups and received intraperitoneal injections of 5% dextrose solution, TP‐lipo, vehicle liposomes, and free TP at a dose of 0.5 mg/kg every other day for a total of 11 doses. The body weight of each mouse was monitored every 3 days. A total of 24 h after the final injection, blood samples were collected for serological assays, while major organs were harvested for H&E staining.

### 4.15 Statistical Analysis

All statistical comparisons, except for survival analysis, were carried out with one‐way ANOVA, utilizing GraphPad Prism 10 Software. Results are reported as mean ± standard error of the mean (SEM). A *p* value < 0.05 was considered significant. Survival curves were generated using the Kaplan–Meier method and compared using the log‐rank test. Pairwise comparisons were adjusted for multiple testing using the Bonferroni correction. A corrected *p* value < 0.05 was considered statistically significant. Univariate Cox proportional hazards regression analysis was performed to estimate HRs and their 95% CIs.

## Author Contributions

Lirui Tang conducted the investigation, analyzed the data, and drafted the manuscript. Yi Lin and Xueqin Jiang performed the experiments and analyzed the data. Xiaohe Tian and Giuseppe Battaglia critically revised the manuscript. Yuquan Wei conceptualized the study. Wenshuang Wu provided resources and supervised the study. Xuemei He validated the data and revised the manuscript. Xiawei Wei conceptualized the study and critically revised the manuscript. All authors have read and approved the final manuscript.

## Funding

This work was supported by the Innovative Drug Research and Development National Science and Technology Major Project (2025ZD1804200, Y.W.).

## Ethics Statement

Sichuan University's Institutional Animal Care and Use Committee gave approval for all animal studies (ethical approval number: 20250313042).

## Conflicts of Interest

Yuquan Wei is an editorial board member of MedComm; however, he was not involved in the peer review or editorial processing of this manuscript. The other authors declare no conflicts of interest.

## Supporting information




**Supporting File 1**: mco270977‐sup‐0001‐SuppMat.docx

## Data Availability

The data that support the findings of this study are available from the corresponding author upon reasonable request.
