# Supplementary material for: Tripterine Liposome Exerts Antitumor and Immunomodulatory Effects in Pancreatic Ductal Adenocarcinoma
Source: MedComm (2020). 2026 Sep 20;7(10):e70977. doi: 10.1002/mco2.70977 (PMC13591019; doi:10.1002/mco2.70977)
Supplement: Supplementary file 1 [file MCO2-7-e70977-s001.docx]

**Supplemental**

**Tripterine Liposome Exerts Antitumor and Immunomodulatory Effects in Pancreatic Ductal Adenocarcinoma**

Lirui Tang^1, #^, Yi Lin^1, #^, Xueqin Jiang^2, #^, Xiaohe Tian^3^, Giuseppe Battaglia^4^, Yuquan Wei^1^, Wenshuang Wu^5, *^, Xuemei He^1, ⁎^, Xiawei Wei^1, ⁎^

^1^ Laboratory of Aging Research and Cancer Drug Target, State Key Laboratory of Biotherapy and Cancer Center, National Clinical Research Center for Geriatrics, West China Hospital, Sichuan University, Chengdu 610041, China.

^2^ Department of Biotherapy, Cancer Center and State Key Laboratory of Biotherapy, West China Hospital, Sichuan University, Chengdu 610041, China

^3^ Functional and Molecular Imaging Key Laboratory of Sichuan Province, Huaxi MR Research Centre (HMRRC), Department of Radiology, Chengdu 610041, China

^4^ Institute for Bioengineering of Catalonia (IBEC), The Barcelona Institute of Science and Technology, Barcelona, Spain

^5^ Division of Thyroid Surgery, Department of General Surgery and Laboratory of Thyroid and Parathyroid Disease, Frontiers Science Center for Disease-related Molecular Network, West China Hospital, Sichuan University, Chengdu 610041, China.

^⁎^Corresponding authors: Wenshuang Wu (wenshuang_wu@163.com), Xuemei He ([hexuemei1116@126.com](mailto:hexuemei1116@126.com)), Xiawei Wei (xiaweiwei@scu.edu.cn)

^#^ These authors contributed equally.

**Figure S1**

**Figure S1.** The gating strategy for the polarization of macrophages in peritoneal lavage fluid from mice. Cells were sequentially gated for singlets, live cells, CD45⁺ leukocytes, CD11b⁺F4/80⁺ macrophages, and finally CD206^-^MHC-II^+^ M1 macrophages and CD206^+^MHC-II^-^ M2 macrophages. Representative plots from *in vivo* samples are shown; the same gating strategy was used for *in vitro* samples, but the specific gate positions and ranges varied.

**Table S1.** The flow cytometry antibodies used in this research.

| Antibody | Source | Cat. No. |
| --- | --- | --- |
| BD Horizon™ RB705 Hamster Anti-Mouse CD3e | BD Biosciences, USA | 570560 |
| Brilliant Violet 510™ anti-mouse CD4 Antibody | BioLegend, USA | 100519 |
| PE anti-mouse CD8a Antibody | BioLegend, USA | 100708 |
| Brilliant Violet 421™ anti-mouse IFN-γ Antibody | BioLegend, USA | 505830 |
| Alexa Fluor® 647 anti-human/mouse Granzyme B Antibody | BioLegend, USA | 515406 |
| BD OptiBuild™ RB705 Rat Anti-Mouse CD45 | BD Biosciences, USA | 505291 |
| FITC anti-mouse/human CD11b Antibody | BioLegend, USA | 101206 |
| APC anti-mouse F4/80 Antibody | BioLegend, USA | 123116 |
| PE anti-mouse CD206 (MMR) Antibody | BioLegend, USA | 141706 |
| Brilliant Violet 421™ anti-mouse I-A/I-E Antibody | BioLegend, USA | 107632 |
| PE anti-mouse CD4 Antibody | BioLegend, USA | 100512 |
| Brilliant Violet 510™ anti-mouse CD8a Antibody | BioLegend, USA | 100752 |
| PE/Cyanine7 anti-mouse IFN-γ Antibody | BioLegend, USA | 505826 |
